# Novel Indigenous Probiotic *Lactobacillus reuteri* Strain Produces Anti-biofilm Reuterin against Pathogenic Periodontal Bacteria

**DOI:** 10.1055/s-0041-1731591

**Published:** 2021-07-24

**Authors:** Armelia Sari Widyarman, Citra Fragrantia Theodorea

**Affiliations:** 1 Department of Microbiology, Faculty of Dentistry, Trisakti University, West Jakarta, Indonesia; 2 Department of Oral Biology, Faculty of Dentistry, Universitas Indonesia, Indonesia

**Keywords:** biofilm, *Lactobacillus reuteri*, *Porphyromonas gingivalis*, reuterin, *Treponema denticola*

## Abstract

**Objective** The aim of this study was to evaluate the effect of reuterin produced by a novel probiotic strain of *Lactobacillus reuteri* against periodontal biofilms.

**Materials and Methods** *L. reuteri* LC382415 (an indigenous Indonesian strain) was cultured in Man, Rogosa, and Sharpe (MRS) agar in anaerobic conditions for 24 hours. To isolate reuterin, *L. reuteri* was suspended in 300-mM glycerol in MRS broth and incubated under anaerobic conditions for 3 hours, and the supernatant fraction was filtered. The presence of reuterin was confirmed by sodium dodecyl sulfate–polyacrylamide gel electrophoresis (SDS-PAGE), and its concentration was determined. The effect of reuterin on *Porphyromonas gingivalis* ATCC 33277 and *T. denticola* ATCC 35405 biofilms was evaluated using biofilm assays. Biofilms were formed by incubating bacteria in 96-well microplates for 48 hours. A dose-dependent experiment was performed with reuterin concentrations of 12.5, 25, 50, and 100 μg/mL on biofilms. The inhibitory effect was measured at 1, 3, 6, and 24 hours. The biofilm masses were measured at 490 nm. Statistical analysis was using one-way ANOVA.

**Results** The SDS-PAGE assay confirmed the presence of reuterin (52 kDa) in the culture supernatant of the *L. reuteri* strain. Reuterin in a concentration as low as 12.5 μg/mL significantly inhibited single- and mixed-species biofilms ( *p* < 0.05).

**Conclusions** This is the first study to demonstrate the promising effect of reuterin isolated from *L. reuteri* LC382415 against periodontal bacteria. Further studies are warranted to explore the mechanism of this active component.

## Introduction

Periodontitis is considered one of the most prevalent diseases worldwide and the main cause of adult tooth loss. 1 It is also associated with systemic pathologies, such as cardiovascular disease, obesity, and diabetes. 2 The etiologic agents of periodontal disease are pathogenic dental plaque biofilms. 3 Under certain conditions, these biofilms harbor gram-negative bacterial pathogens such as *Porphyromonas gingivalis* and *Treponema denticola* , which are closely associated with periodontitis. 4 These bacteria produce virulence factors that evoke an exacerbated immune-inflammatory response, leading to the destruction of periodontal tissues. Traditionally, periodontitis is treated by mechanical debridement of dental plaque combined with chemical plaque control measures. However, periodontal therapy is costly and may not be sufficiently effective. Therefore, numerous studies have explored alternative therapies for periodontal disease. 5 6

Probiotics have been shown to provide numerous benefits as in treatments for various human diseases, including allergic diseases (atopic dermatitis), bacterial vaginosis, urinary tract infections, and gastrointestinal disorders, and as preventives of dental caries. 7 8 9 10 According to the World Health Organization, the term “probiotics” refers to “live microorganisms (that) when administered in adequate amounts confer a health benefit on the host.” 11 Probiotics have a long history of use in health promotion and are generally considered safe. 12 *Lactobacillus* spp. and *Bifidobacterium* spp. are commonly used probiotic bacteria. 13 Previous studies have attempted to examine the role of probiotics in various oral diseases, including periodontitis. 14

*Lactobacillus* spp. produce antimicrobial substances, such as bacteriocins. Laboratory strains of *Lactobacillus reuteri* are known to produce an antimicrobial compound called reuterin, which is a low molecular class of bacteriocin. 15 However, there are no studies on the antibacterial effect of clinical strains of reuterin isolated from *L. reuteri,* particularly in periodontitis. To fill this research gap, this first study attempted to investigate the antibacterial properties of *L. reuteri* LC382415 against pathogenic periodontal bacteria. Moreover, we isolated reuterin and examined its activity against pathogenic periodontal biofilms.

## Materials and Methods

### Isolation and Purification of Reuterin

Previous study done by Widyarman and colleagues in 2018 succeeded in isolating an indigenous Indonesian probiotic *L. reuteri* strain (LC382415) from a healthy young adult after a comprehensive screening assay. 16 At first we compare the presence of reuterin isolated from *L. reuteri* LC382415 and *L. reuteri* ATCC 55730 (as a control) with the molecular weight 52 kDa.

In this *in vitro* experimental study, strain *L. reuteri* LC382415 was inoculated on de Man, Rogosa, and Sharpe (MRS) agar plate and incubated in anaerobic conditions at 37°C for 72 hours. This colony was then harvested via centrifugation at 4,000 × g for 10 minutes and washed twice with phosphate-buffered saline (Oxoid) (pH 7.4).

Reuterin was isolated following a protocol used by Chen et al and Spinler et al with some modifications. 17 18 In brief, an *L. reuteri* LC382415 culture was harvested at 1.5 × 10 CFU/mL and resuspended in 5 mL of 300-mM glycerol in MRS broth. This inoculum was incubated under anaerobic conditions at 37°C for 3 hours. After fermentation, the cells were collected via centrifugation at 4,000 × g for 10 minutes. The supernatant fraction was filtered using a filter membrane (pore size: 0.22 μm; Millipore) and stored at 4°C. The presence of reuterin in the medium was determined by analyzing its molecular weight using sodium dodecyl sulfate–polyacrylamide gel electrophoresis (SDS-PAGE) method. Four concentrations of reuterin (12.5, 25, 50, and 100 μg/mL) were prepared using the Bradford method. 19 The Bradford assay showed a mean reuterin concentration of 208.06 μg/mL.

### Confirmation of Reuterin by SDS-PAGE

SDS-PAGE was used to confirm the presence of reuterin in the samples using a protocol published by Costas with modifications. 20 The polyacrylamide gel was prepared by assembling gel cassettes using clean glass plates. We combined 30 mL of acrylamide stock solution, 22.5 mL of separating gel buffer, and 36.15 mL of deionized water in a side-arm flask and degassed the solution using a vacuum pump for 3 minutes. While the solution was being gently swirled to ensure adequate mixing, 0.9 mL of 10% (w/v) SDS stock was added, along with 0.45 mL of 10% ammonium peroxydisulfate and 45 μL of tetramethylethylenediamine (TEMED). Two gels were poured, delivering 10 mL volumes of acrylamide/buffer mixture. When the mixture reached 30 mm from the top of the plates, polytetrafluoroethylene (PTFE)-coated combs were carefully inserted, projecting 35 mm into the gel cassettes. We prepared a stacking gel mix in a side-arm flask for two gels (20 mL). This gave a final concentration of 5% acrylamide. The mixture was subsequently degassed, and 200 μL of 10% (w/v) SDS was then added, along with 100 μL of 10% (w/v) ammonium peroxydisulfate and 20 μL of TEMED. PTFE combs were inserted in 15 to 20 wells 25 mm into the assembly, leaving 10 mm for the stacking gel. The gel was left to set for 1 hour. The combs were then removed, and the sample wells were washed thoroughly with deionized water and filled with reservoir buffer.

To prepare the sample, we added 10 μL of native buffer into 30 μL of sample proteins and heated the mixture in a thermoblock machine (BioSan) at 100°C for 5 minutes. After that, 20 μL of the protein extract was loaded into each well using a micropipette. The gels were then removed from the assembly/pouring stand, placed in a running apparatus, and filled with tank buffer. The gels were electrophoresed at a constant current of 80 mA (50 V, 30 W) and a constant temperature of 15°C for 2 hours. Coomassie Blue staining solutions were then added. 20 Bradford assays were performed to visualize the reuterin bands using a Coomassie Plus Bradford Assay Kit (Thermo Fisher Scientific) according to the manufacturer’s instructions. A standard curve was used to determine the protein concentration of each sample. Bovine serum albumin (Thermo Fisher Scientific) was used as a control sample.

### Reuterin on Pathogenic Oral Bacterial Biofilms

The activity of reuterin isolated from *L. reuteri* LC382415 against oral pathogenic bacterial biofilms was assessed using monospecies and dual-species biofilms of *P. gingivalis* ATCC 33277 and *T. denticola* ATCC 35405. The bacteria were cultured on Brain Heart Infusion (BHI) (Sigma Aldrich) broth using the anaerobic GasPak jar system and incubated at 37°C for 48 hours. 21 For monospecies biofilms, 200 μL of 10 ^7^ -CFU/mL *P. gingivalis* or *T. denticola* inoculum was added to 96-well plates. For dual species biofilms, 100 μL of 10 ^7^ -CFU/mL *P. gingivalis* inoculum and 100 μL of 10 ^7^ -CFU/mL *T. denticola* inoculum were added into 96-well plates (NEST). Both monospecies and dual species inocula were incubated under anaerobic conditions at 37°C for 48 hours to form biofilms. Four different concentrations of reuterin (12.5, 25, 50, and 100 μg/mL) were then added to the biofilms to evaluate its antibacterial activity. The effects were assessed at 1, 3, 6, and 24 hours. 22 23 Chlorhexidine 0.2% was used as a positive control. Wells containing biofilms without reuterin were used as negative controls.

### Biofilm Examination

The first examination was conducted using crystal violet analysis. The next examination was conducted using total plate count for enumeration of CFUs.

Crystal violet (0.5% w/v) was added to all wells, incubated for 15 minutes, and then removed. Absolute ethanol (200 μL) was also added to all wells to dissolve remaining crystal violet-biofilm complex. Dissolved crystal violet within each well was measured and considered as biofilm mass.

After crystal violet staining procedure, the photos of biofilm mass were observed by Carl Zeiss Primovert Inverted Microscope (Germany) at a 40x objective. Furthermore the absorbance measurements were performed using a microplate reader (SAFAS) at a wavelength of 595 nm. All treatments were performed in independently triplicate experiments.

For the total plate count, after biofilms were washed with PBS and dried at room temperature, the formed biofilm layer at the base of well was collected and diluted 10-fold. Ten microliters of the dilution solution with concentrations of 10 ^−4^ to 10 ^−7^ were poured on each MRS agar medium plate and were incubated anaerobically at 37°C (2 × 24 h), and then the CFU count was performed manually.

### Standard Curve for Absorbance and Biofilm Quantification

To quantify absorbance (optical density) for bacterial concentration measurement, a standard curve was used to demonstrate the relationship between optical density and colony count, *P. gingivalis* and *T. denticola* inocula at 1 Log10 CFU/mL were diluted in BHI (Sigma Aldrich) broth in a 1:10 dilution series to achieve six dilutions (10–10) (►Supplementary Fig. S1, available in the online version only). Biofilms were grown under static conditions in sterile 96-well microplates, with each well containing 200 μL, at 37°C for 24 hours. Upon removal from incubation, the biofilms were heat fixed and stained with crystal violet. The absorbance was measured at a 595-nm excitation wavelength. 24

### Statistical Analysis

The obtained results were statistically analyzed using one-way analysis of variance (ANOVA) to assess differences in biofilm reduction. The level of significance was set to *p* < 0.05. The Shapiro-Wilk test was used to assess normality, and Levene’s test was used to assess the homogeneity of variance. The statistical analysis was performed using IBM SPSS Statistics version 20 (IBM Corp.) for Windows. 25

## Results

### Isolation of Reuterin, SDS-PAGE, and Bradford Assay

**Fig. 1:**
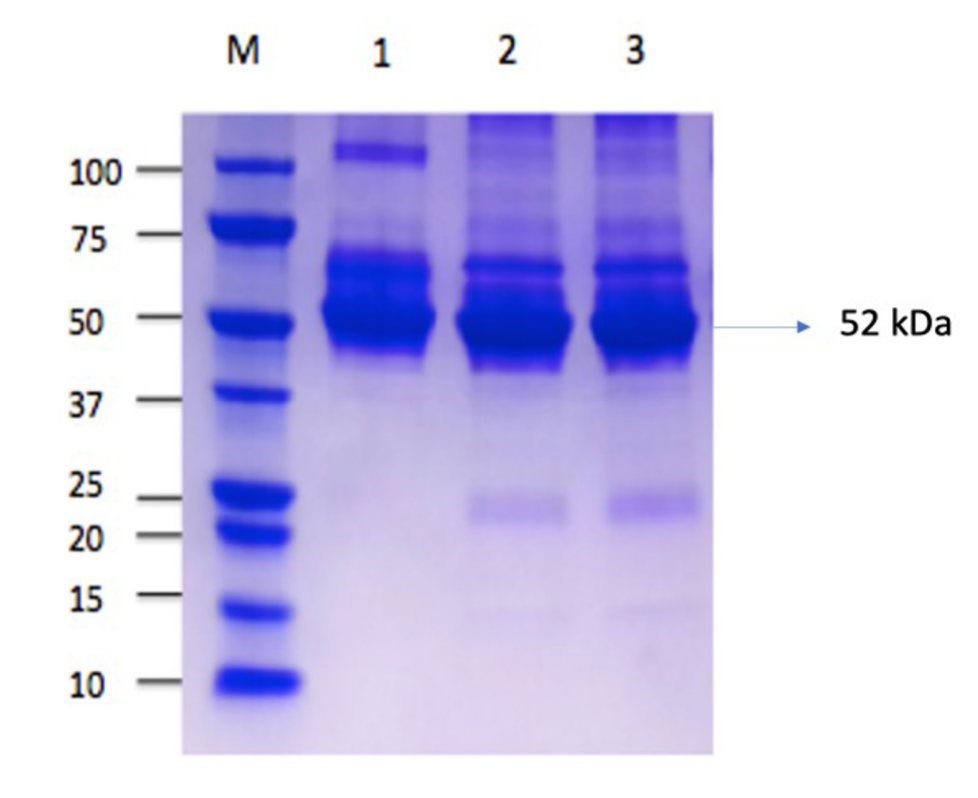
Reuterin isolate from *L. reuteri* in 12% polyacrylamide gel. M, marker protein (molecular weight: 15–100 kDa); bands 1: reuterin isolate from *L. reuteri* ATCC 55730; bands 2–3: reuterin isolate from Indonesian LC382415 strain (52 kDa).

The presence of reuterin isolated from *L. reuteri* LC382415 and ATCC 55730 is shown in Fig. 1 . SDS-PAGE of the LC382415 culture supernatants showed a 52-kDa band, confirming the presence of reuterin. 26

### Activity of Reuterin against Pathogenic Periodontal Biofilms

**Fig. 2:**
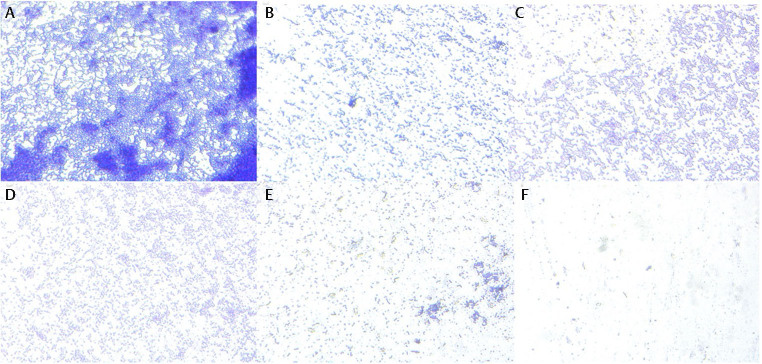
Inverted optical images of *Porphyromonas gingivalis* biofilms after treatment with various concentrations of reuterin and control: ( **A** ) Treated with bacterial culture medium as a negative control; ( **B** ) Treated with 12.5 µg/mL reuterin; ( **C** ) Treated with 25 µg/mL reuterin; ( **D** ) Treated with 50 µg/mL reuterin; ( **E** ) Treated with 100 µg/mL reuterin; ( **F** ) Treated with 0.2% chlorhexidine as a positive control. The biofilms were observed using Carl Zeiss Primovert Inverted Microscope at a 40x objective.

**Fig. 3:**
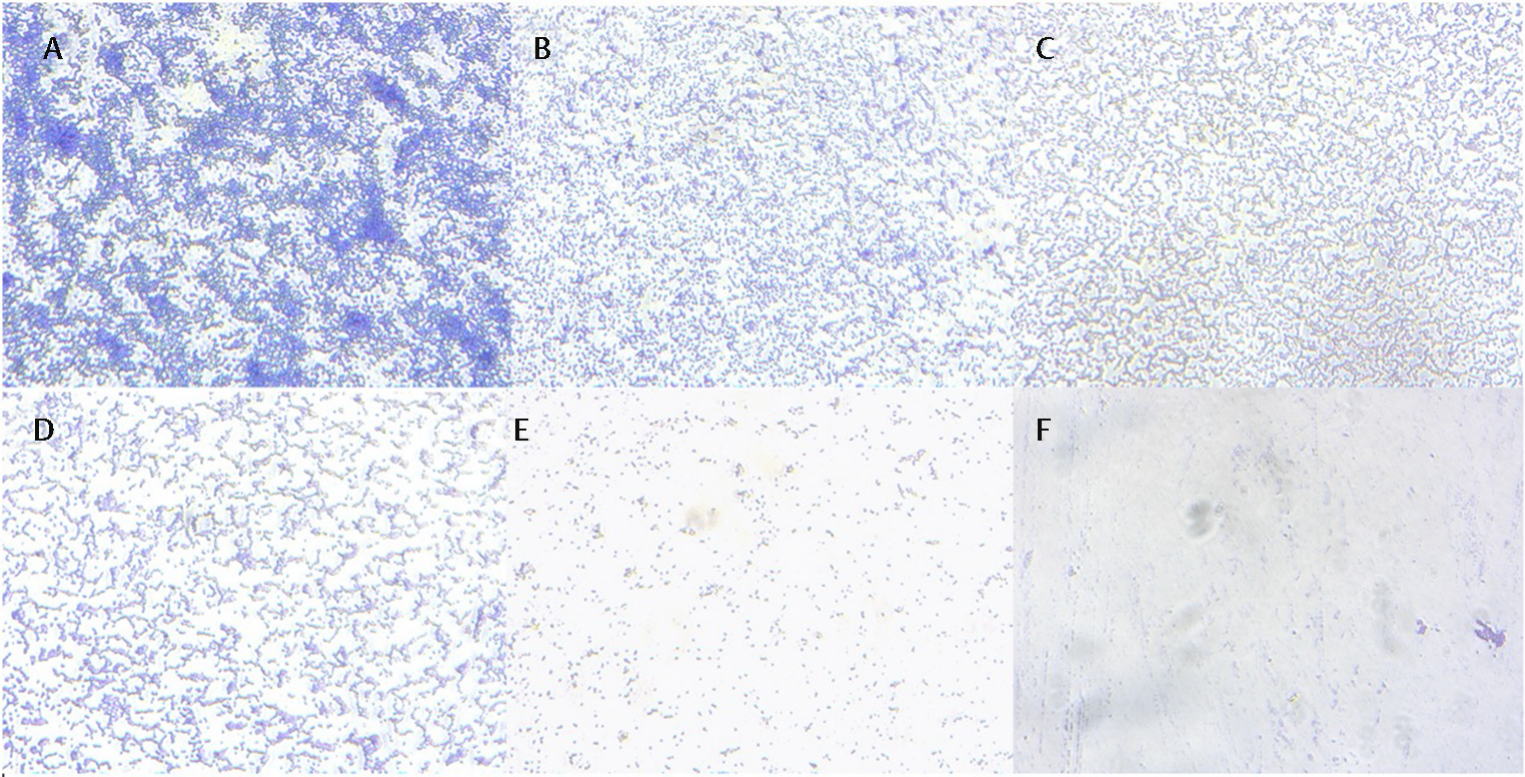
Inverted optical images of *Treponema denticola* biofilms after treatment with various concentrations of reuterin and control: ( **A** ) Treated with bacterial culture medium as a negative control; ( **B** ) Treated with 12.5 µg/mL reuterin; ( **C** ) Treated with 25 µg/mL reuterin; ( **D** ) Treated with 50 µg/mL reuterin; ( **E** ) Treated with 100 µg/mL reuterin; ( **F** ) Treated with 0.2% chlorhexidine as a positive control. The biofilms were observed using Carl Zeiss Primovert Inverted Microscope at a 40x objective.

The photographs of the biofilm lining 96-well plates of reuterin against each monospecies *P. gingivalis* and *T. denticola* were taken after staining with crystal violet. The biofilms were observed using Carl Zeiss Primovert Inverted Microscope at a 40x objective ( Figs. 2 and 3 ).

**Fig. 4:**
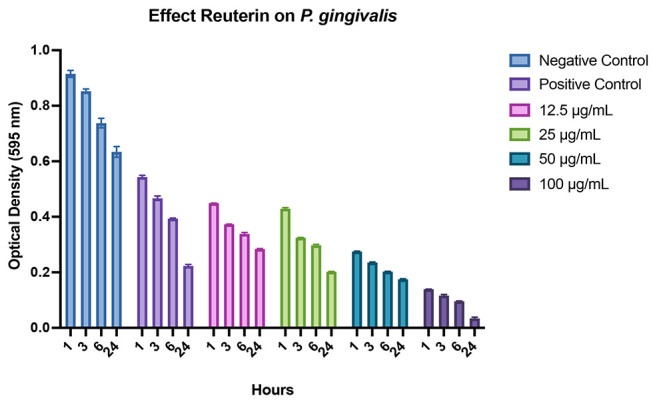
Graphs showing the inhibition of *Porphyromonas gingivalis* biofilm growth after treatment with reuterin at different concentrations (μg/mL) and incubation times, as well as comparisons with positive and negative controls. *P. gingivalis* treated with bacterial culture medium as a negative control; *P. gingivalis* treated with 0.2% chlorhexidine as a positive control. Each graph shows the average of independently triplicate experiments.

**Fig. 5:**
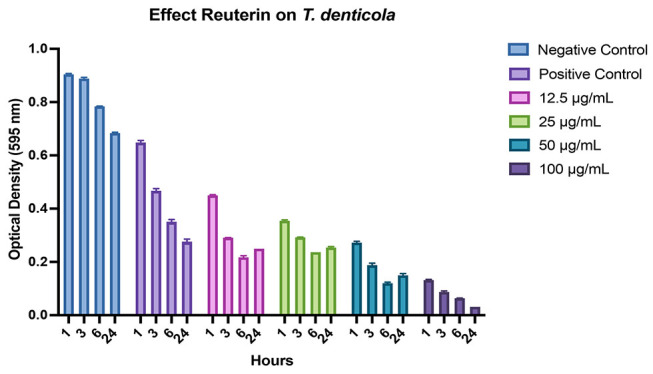
Graphs showing the inhibition of *Treponema denticola* biofilm growth after treatment with reuterin at different concentrations (μg/mL) and incubation times, as well as comparisons with positive and negative controls. *Treponema denticola* treated with bacterial culture medium as a negative control; *Treponema denticola* treated with 0.2% chlorhexidine as a positive control. Each graph shows the average of independently triplicate experiments.

**Fig. 6:**
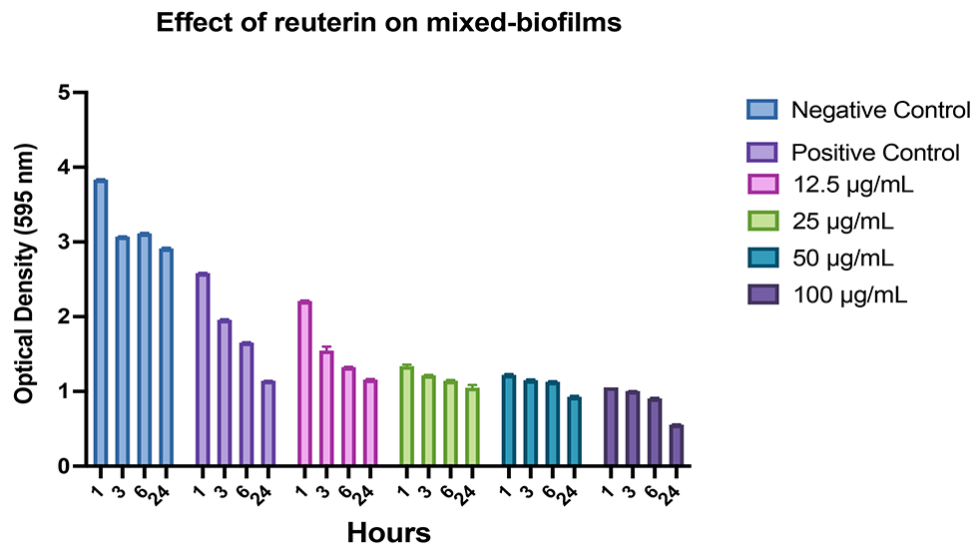
Graphs showing the inhibition of mixed-biofilm growth after treatment with reuterin at different concentrations (μg/mL) and incubation times, as well as comparisons with positive and negative controls. Each graph shows the average of independently triplicate experiments.

The biofilm assay by using crystal violet showed that reuterin significantly inhibited monospecies *P. gingivalis* and *T. denticola* in dose dependent activity and incubation time. Reuterin in a concentration as low as 12.5 μg/mL significantly inhibited single- and mixed-species biofilms ( *p* <0.05). The most effective of reuterin concentration in the monospecies biofilm of *P. gingivalis* and *T. denticola* was 100 μg/mL due to which it can inhibit up to 50 and 77% reduction, respectively, compared with negative control ( Figs. 4 and 5 ) in 1 hour and 100 μg/mL reuterin concentration showed significantly inhibited monospecies of those bacteria in prolonged incubation time compared with negative control. Moreover, reuterin exhibited a significant dose-dependent activity against mixed-species *P. gingivalis* and *T. denticola* biofilms in a concentration as low as 12.5 μg/mL ( Fig. 6 ) within 3 hours. Incubation for up to 24 hours with higher concentrations (25–100 μg/mL) showed an even more significant anti-biofilm activity.

**Fig. 7:**
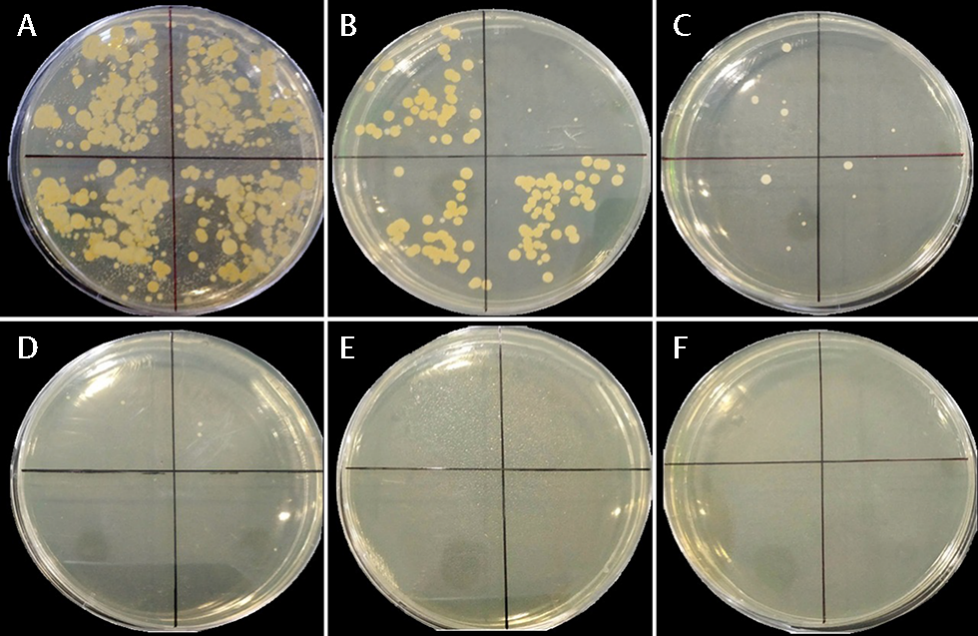
CFU counts at various incubation times were done to determine the anti-biofilm activity of concentration-dependent reuterin. ( **A** ) *Porphyromonas gingivalis* treated with bacterial culture medium as a negative control; ( **B** ) *P. gingivalis* treated with 12.5 µg/mL reuterin; ( **C** ) *P. gingivalis* treated with 25 µg/mL reuterin; ( **D** ) *P. gingivalis* treated with 50 µg/mL reuterin; ( **E** ) *P. gingivalis* treated with 100 µg/mL reuterin; ( **F** ) *P. gingivalis* treated with 0.2% chlorhexidine as a positive control.

**Fig. 8:**
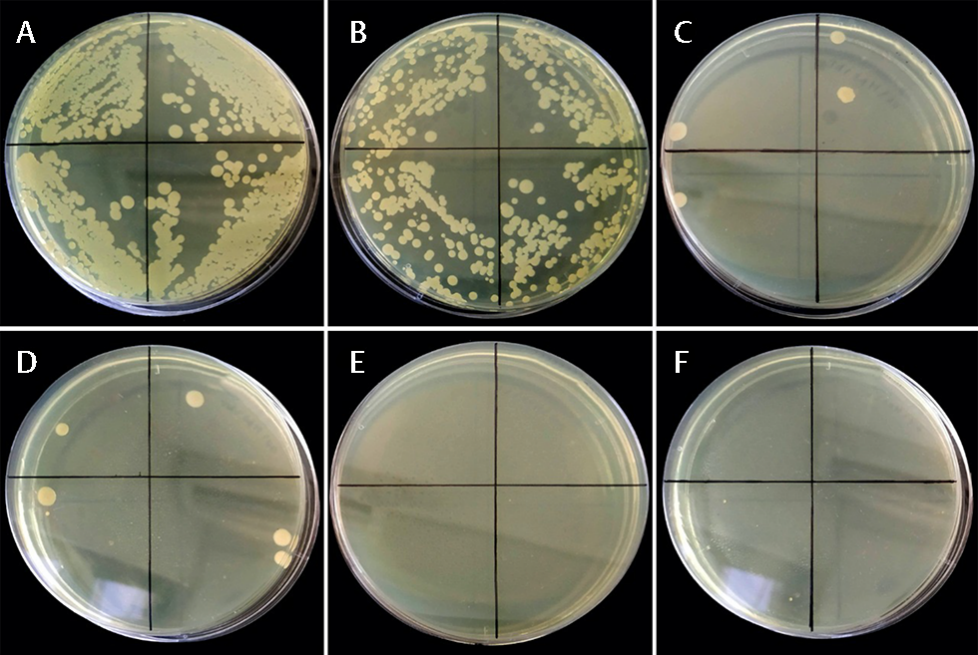
CFU counts at various incubation times were done to determine the anti-biofilm activity of concentration-dependent reuterin. ( **A** ) *Treponema denticola* treated with bacterial culture medium as a negative control; ( **B** ) *Treponema denticola* treated with 12.5 µg/mL reuterin; ( **C** ) *Treponema denticola* treated with 25 µg/mL reuterin; ( **D** ) *Treponema denticola* treated with 50 µg/mL reuterin; ( **E** ) *Treponema denticola* treated with 100 µg/mL reuterin; ( **F** ) *Treponema denticola* treated with 0.2% chlorhexidine as a positive control.

In the total plate count analysis of CFUs, the presence of reuterin isolated from *L. reuteri* LC382415 on each monospecies biofilm of *P. gingivalis* and *T. denticola* showed anti-biofilm activity of concentration-dependent reuterin at various incubation time ( Figs. 7 and 8 ).

Overall, there was effect of reuterin to decrease each monospecies biofilm of *P. gingivalis* and *T. denticola* in dose-dependent activity and incubation time.

## Discussion

Periodontal diseases represent a significant health burden, with an estimated annual cost of USD 8 billion, particularly in Malaysia. 27 Therefore, considerable efforts have been made to explore new therapeutic strategies against periodontal bacteria. In this study, we found that novel clinical isolates of an indigenous Indonesian *L. reuteri* strain show promising properties against periodontal pathogens through a reuterin-based mechanism. Importantly, we also found that reuterin can inhibit biofilm formation of periodontal bacteria.

The antimicrobial activity of probiotics is associated with the production of organic acids, peptides (bacteriocins), carbon dioxide, hydrogen peroxide, ethanol, and diacetyl. 28 Treatment strategies based on probiotics exert antibacterial activities through two mechanisms: (1) inhibition of specific organisms by interfering with adhesion, colonization, and biofilm formation and (2) inhibition of pathogen growth via various substances, such as organic acids, hydrogen peroxide, and bacteriocins. 29 Through these mechanisms, probiotics can inhibit the growth of dental plaque biofilms, which is in line with the findings of this study. Haukioja, in 2010 reported that patients with gingivitis, periodontitis, and pregnancy gingivitis locally treated with a culture supernatant of an *L. acidophilus* strain showed significant recovery. 30 Other studies examining *L. reuteri* strains and *L. brevis* have also reported improved gingival health, as measured by decreased gum bleeding and inflammation. 31 32 33

In this study, we also assessed reuterin efficacy as antibiofilm agent against mixed-species biofilm from two periodontitis-causing bacteria. This is done to mimic the condition of major pathogens that cause periodontitis *in vitro* . It can be inferred from Fig. 5 that higher concentration of reuterin better reduces the biofilm mass concentration. It can be postulated that reuterin has the property of antimicrobial effects by inhibiting the production of bacterial ribonucleotide reductase. This is an enzyme that catalyzes the first step in DNA synthesis via competition (HPA-dimer) with ribonucleotides for binding sites or via reaction (3-HPA) with the unstable sulfhydryl groups of ribonucleotide reductase or thioredoxin. This inhibitory activity may explain the broad-spectrum activity of reuterin against periodontal bacteria. 34 Thus, the higher concentration of the reuterin may give higher efficacy in reducing bacterial biofilm mass. This study provides encouraging evidence for the treatment of periodontal disease using probiotic-derived product, such as reuterin. Further studies are warranted to explore the effect of reuterin *in vivo* .

## Conclusions

This study is the first to demonstrate the activity of reuterin derived from clinical isolates of *L. reuteri* against monospecies and dual-species periodontal bacterial biofilms. The findings could lead to novel therapeutic strategies against periodontitis, benefiting patients worldwide.
